# The long non-coding RNA *CidecAS* regulates hepatocyte lipid metabolism via the alpha-1 subunit of Na^+^/K^+^-ATPase

**DOI:** 10.3389/fnut.2026.1809132

**Published:** 2026-05-12

**Authors:** Lin Yu, Siqi Liu, Yang Xiao, Jiale Wang, Xingzhen Yang, Qiuchen Cheng, Qiuhua Li, Dan Yin, Yuehui Liang, Xue Liang, Menglong Hou, Jingsu Yu, Yixing Li, Lei Zhou, Yunxiao Liang

**Affiliations:** 1 Department of Gastroenterology, Guangxi Academy of Medical Sciences, The People’s Hospital of Guangxi Zhuang Autonomous Region, Nanning, China; 2 Guangxi Key Laboratory of Animal Breeding, Disease Control, and Prevention, College of Animal Science and Technology, Guangxi University, Nanning, China

**Keywords:** ATP1a1, lipid metabolism, *lnc-CidecAS*, MAFLD, sodium-potassium pump

## Abstract

**Introduction:**

The rising prevalence of metabolic-associated fatty liver disease (MAFLD) poses a serious public health threat, while long non-coding RNAs, key regulators of hepatic lipid metabolism, are closely linked to its development and progression. This study identified a novel MAFLD-associated antisense lncRNA, *lnc-CidecAS*, aiming to characterize its molecular structure and elucidate its regulatory role in hepatic lipid metabolism.

**Methods:**

The sequence characteristics and coding potential of *lnc-CidecAS* were determined using RACE technology and flag-tagged expression vectors. Overexpression in AML12 hepatocytes was conducted to assess its effects on lipid metabolism-related genes and extracellular triglyceride (TG) levels. Both aged mice and HFD-induced obesity models were utilized for *in vivo* validation. Physiological parameters from blood, liver, and muscle tissues were measured after adeno-associated virus-mediated delivery of *lnc-CidecAS* to evaluate systemic lipid metabolism. Mechanistically, ChIRP-MS was employed to identify *lnc-CidecAS* interacting proteins, and the functional interaction with ATP1a1 was confirmed through siRNA knockdown and enzymatic activity assays.

**Results:**

*Inc-CidecAS* was primarily localized in the cytoplasm. Its overexpression in AML12 cells significantly reduced extracellular TG levels while upregulated key lipid metabolism genes (*AMPK*, *ATGL*, *HSL*, *CPT1* and *ACOX1*). *In vivo*, *lnc-CidecAS* expression decreased under fasting conditions, declined with age, and showed a negative correlation with blood lipid levels. Overexpression of *lnc-CidecAS* reduced body fat and serum lipid concentrations in mice. In this HFD-induced obesity model, hepatic-specific overexpression of *lnc-CidecAS* markedly alleviated fat deposition in the liver and muscle, concurrently lowering serum TG and total cholesterol. Mechanistic studies revealed that *lnc-CidecAS* binds to ATP1a1, enhancing its gene expression and enzymatic activity, thereby promoting lipid metabolism.

**Discussion:**

Our study reveals the regulatory role of *lnc-CidecAS* in hepatocyte lipid metabolism, and reveals its molecular mechanism via interaction with ATP1a1, identifying a novel *lnc-CidecAS*–ATP1a1 regulatory axis. This discovery expands our understanding of how lncRNAs cooperate with proteins to regulate cellular metabolism. Consequently, targeting this pathway provides a theoretical foundation for developing precise therapies against MAFLD and related metabolic disorders.

## Graphical abstract

**Figure:**
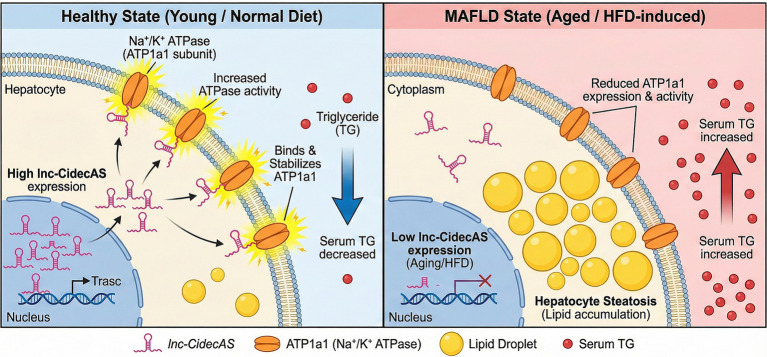

## 1 Introduction

As the central organ, the liver is primarily responsible for detoxification and glycolipid metabolism. Over the past two decades, shifts in lifestyle and dietary habits have contributed to the rise of metabolic-associated fatty liver disease (MAFLD). Its global prevalence ranges from 25.5 to 32.4%, thereby posing a significant public health concern (1, 2). The primary diagnostic criterion for MAFLD is excessive accumulation of liver lipids in the absence of significant alcohol intake. The pathological process of MAFLD primarily involves an imbalance in lipid synthesis, oxidation, and transport (3, 4). Hepatic fibrosis and cirrhosis represent advanced stages in the MAFLD-MASH progression cascade, which, if left untreated, elevates the risk for developing hepatocellular carcinoma (5, 6). Approximately 20%–30% of MAFLD cases will reportedly progress to MASH (7, 8). Current treatment for MAFLD primarily involves lifestyle interventions (diet and exercise) or the use of drugs, such as PPAR agonists and FXR receptor agonists (9, 10). Since the Food and Drug Administration has not yet approved any universal specific medication for MAFLD (11, 12), few drugs have received only limited approval for specific subtypes, such as MASH with fibrosis (13). Consequently, identifying novel therapeutic targets is critical for preserving hepatic lipid homeostasis and promoting overall physiological well-being.

Long non-coding RNAs (lncRNAs) are distinguished from other RNAs by their length, which extends beyond 200 nucleotides, and are characterized as non-coding. Accumulating experimental data have demonstrated their association with diverse biological processes, including cellular development and cell fate specification (14), immunological responses (15), especially the release of inflammatory factors (16), stress responses (17, 18), metabolic regulation, and lipid deposition (19, 20). Genetic variants also modulate lncRNA function and their interactions with RNA-binding proteins (RBPs), adding another layer of complexity to liver metabolic control (21). Molecularly, lncRNAs govern gene regulation via roles in signal transduction, epigenetics, and transcriptional and post-transcriptional control. Moreover, lncRNAs can alter the state of chromatin by recruiting chromatin-modifying complexes, such as DNA methyltransferases and histone modification enzymes, to exert epigenetic control. For instance, the lncRNA *Xist* mediates silencing of the X chromosome (22, 23). In transcriptional regulation, lncRNAs can act as scaffold molecules to bind transcription factors (such as p53, SMAR1, and the HIV promoter) or RNA polymerases, influencing the activity of promoters or enhancers (24, 25). Post-transcriptional regulation is even more diverse, as lncRNAs can act as “sponges” of miRNAs, competitively binding target miRNAs and relieving inhibition of target mRNAs (19, 26), and can also bind complementarily to mRNAs, affecting splicing, stability, and translation (27). Additionally, lncRNAs can regulate the localization and activity of RNA-interacting proteins. A case in point is the lncRNA *ZFAS1*, which functions as a transcriptional repressor to modulate lung cancer pathogenesis (28). For instance, the non-conserved human lncRNA *hLMR1* binds the splicing factor PTBP1 to promote transcription of cholesterol biosynthetic genes, thereby regulating hepatic cholesterol metabolism (29). LncRNAs could alter the transcriptional output of the Wnt and NF-κB pathways by regulating their key molecules (30, 31). In the context of lipid metabolism diseases, lncRNAs primarily regulate key pathways involved in lipid synthesis, oxidation, and transport, thereby participating in the initiation and development of MAFLD. For instance, the lncRNA *Gm35585* can inhibit lipid synthesis by activating the PPARα pathway (32), while the lncRNA *AK142643* can upregulate the IGF2 signaling pathway, exacerbating lipid accumulation in hepatocytes (33). In terms of lipid catabolism, SIRT6-associated lncRNAs were reported to improve MAFLD by facilitating fatty acid utilization (34, 35). *LncRHL* ameliorates hepatic steatosis by inhibiting the secretion of very low-density lipoproteins (VLDLs) (36). Regarding lipid transport, the lncRNA *Obr* affects obesity progression by regulating lipid transport across tissues (37). In addition, the lncRNA *Dax1* not only interacts with the transcription factor TFEB to inhibit autophagy, but also directly suppresses activity of LXRα, thereby blocking cholesterol efflux and related lipid transport pathways in macrophages (38, 39). Additionally, lncRNAs can indirectly regulate blood lipid and cholesterol metabolism (40, 41).

In this investigation, a previously uncharacterized lncRNA was discovered among hundreds of differentially expressed lncRNAs in mouse liver. This transcript, designated *lnc-CidecAS*, is situated on the antisense strand of the *Cidec* gene. Expression of *lnc-CidecAS* is significantly reduced during fasting, suggesting involvement in lipid metabolism or other energy metabolic pathways. Given its novelty, the primary objective of this work is to characterize the architecture and spatiotemporal expression dynamics of *lnc-CidecAS*, as well as the molecular mechanisms regulating lipid metabolism.

The findings of this study provide important theoretical evidence to clarify the underlying pathogenesis of MAFLD. Our work thereby offers a rationale for the development of precise, genetically targeted therapies against MAFLD, which could alleviate its global health burden.

## 2 Materials and methods

### 2.1 Animal model and ethics statement

Six-week-old C57BL/6 mice (Tianqin Biotechnology Co., Ltd., Changsha, China) were maintained under standard conditions with a 12-h light/dark cycle, a constant temperature of 22 °C, and free access to food and water. Following a 14-day adaptation period, they were randomly allocated into four distinct dietary regimens. Two of the four groups were maintained on a standard diet (#LAD1000G; Trophic Animal Feed High-tech Co., Ltd., Nantong, China), whereas the other two groups received a high-fat diet (HFD) (60% fat, #TP23520; from the same supplier). Between weeks 8 and 20, we periodically collected blood from mouse tail tip to assess blood glucose and TG levels. Intravenous tail vein injection was used to deliver AAV8-CMV (control) and AAV8-CMV-*lnc-CidecAS* viruses at a dose of 100 μL per mouse, each at a titer of 1.26 × 10^12^ vg/mL (HanBio Co., Ltd., Shanghai, China). Both vectors contained a bovine growth hormone poly(A) signal. Body composition, including body weight and fat mass, was monitored using a small animal body composition analyzer (#QMR23-060H-I; Suzhou Niumag Analytical Instrument Co., Ltd., Suzhou, China). At the study endpoint (week 20), all animals were humanely euthanized. Briefly, a cotton ball saturated with diethyl ether (approximately 5–10 mL) was placed in a 500 mL wide-mouth jar. The mouse was placed inside the jar, and anesthesia was confirmed after approximately 4–6 min by the loss of corneal reflex, pain reflex, slowed respiration, and spontaneous recumbency. Upon reaching surgical anesthesia depth, the mouse was immediately removed and euthanized by cervical dislocation. Death was confirmed by the cessation of respiration and heartbeat, as well as the absence of toe-pinch reflex.

Liver and adipose tissue samples were harvested, promptly placed in 2-mL cryovials (Axygen, Wujiang, China), and immediately stored in an ultralow-temperature freezer (Thermo Scientific, Shanghai, China). All procedures involving mice were performed in compliance with the guidelines outlined in the *Guide for the Care and Use of Laboratory Animals*, and were formally approved by the Institutional Animal Care and Use Committee of Guangxi University (Protocol No. 2020-gxu-253).

### 2.2 RNA extraction and gene quantification

Fresh tissue samples (approximately 100 mg) or cells underwent lysis in TRIzol reagent (#15596-026CN; Tsingke Biotech Co., Ltd., Beijing, China). Homogenization was performed with a TissueLyser® (Qiagen GmbH, Hilden, Germany) for 5 min until a uniform homogenate was achieved. Upon completion of centrifugation at 7,500×g for 6 min, all supernatant was carefully collected into a fresh 2-mL RNase-free tube (Eppendorf SE, Hamburg, Germany) and temporarily stored on ice. RNA isolation was performed with a commercial RNA extraction kit (GenStar®; Beijing Kangrun Chengye Biotechnology Co., Ltd., China). All RNA samples were mixed with Recombinant RNase Inhibitor (0.5 μL/10 μL; #2313Q; Takara Biomedical Technology, Beijing, China), and were either preserved in the ultralow-temperature freezer or immediately used for subsequent cDNA synthesis (#6210A; Takara, from the same supplier). Quantitative polymerase chain reaction (qPCR) was conducted with the Analytik Jena qTOWER 3.0 system (GmbH+Co. KG, Jena, Germany) with 2 × Real Star Green Fast Mixture (GenStar®; Beijing Kangrun Chengye Biotechnology Co., Ltd.) under recommended cycling conditions. All qPCR data were normalized to the housekeeping gene *36b4* (*Rplp0*). Relative expression levels were calculated using the 2^−ΔΔCT^ method, where the expression of each target gene in each sample was first normalized to housekeeping gene and then expressed as a fold change relative to the mean value of the control group.

### 2.3 Nucleocytoplasmic separation and lncRNA sequence acquisition

Mouse liver tissues were lysed and subjected to nuclear/cytoplasmic fractionation using a commercial kit (#21000; Norgen Biotek Corp., Thorold, ON, Canada), with the isolated components stored at −80 °C for future use. Additionally, freshly prepared mouse liver tissue homogenates were utilized for 5′ and 3′ rapid amplification of cDNA ends (RACE) according to the instructions of the SUPERSWITCH™ RACE cDNA Amplification Kit (Beijing Bichenglan Biology Technology Co. Ltd., Beijing, China). Primers can be found in Supplementary material 1.

### 2.4 ChIRP-MS

The chromatin isolation by RNA purification (ChIRP) assay for *lnc-CidecAS* was performed using the Magna ChIRP™ RNA Interactome Kit (#17-10494; Merck KGaA, Darmstadt, Germany). Fresh tissue pieces (approximately 200 mg) were placed in a dish containing 20 mL of ice-cold 1 × PBS. The tissue was minced into small pieces (approximately 1 mm^3^ or smaller) with a clean blade to enhance crosslinking efficiency. Formaldehyde was added to a final concentration of 1%, and crosslinking was performed at room temperature for 10 min with rotation. The reaction was quenched by adding 5 mL of 1.25 M glycine (prepared in DEPC-treated water) and incubation was continued with rotation for an additional 5 min. The cells were collected, washed twice with ice-cold PBS buffer, and pelleted by centrifugation. A set of antisense oligonucleotide probes specific to the *lnc-CidecAS* sequence (see Supplementary material 1) was designed and biotinylated at the 3′ ends using a Biotin 3′ End DNA Labeling Kit (#D3106; Beyotime Biotechnology, Shanghai, China). The probes were divided into two pools based on their odd or even numbering for subsequent experiments. A lacZ probe pool provided by the kit was used as a negative control to exclude non-specific binding signals.

The crosslinked cell pellets were resuspended in Lysis Buffer supplemented with Protease Inhibitor Cocktail III (1:200 dilution) and RNase inhibitor (1:200 dilution). Chromatin was sheared to obtain fragments of 100–500 bp using a water bath sonicator (Q800R, Qsonica, Newtown, CT, United States) at 4 °C with the following settings: 65% power, 15 s ON and 45 s OFF pulse intervals, and a total sonication time of 2 h (corresponding to a total process time of approximately 8 h). After sonication, the lysate was centrifuged at 16,100×g for 10 min at 4 °C, and the supernatant was collected. One milliliter of sheared chromatin lysate was mixed with 2 mL of Hybridization Buffer (containing 15% formamide, protease inhibitor, and RNase inhibitor), and 100 pmol of biotinylated probes (the even and odd probe pools were added separately) was added. Hybridization was performed at 37 °C for 4 h. Following hybridization, 120 μL of pre-washed streptavidin magnetic beads (pre-washed with Lysis Buffer) were added, and the mixture was incubated at 37 °C for an additional 30 min. The beads were washed five times with 1 mL of pre-warmed Wash Buffer (37 °C) supplemented with protease inhibitor; during the first wash, the beads were transferred to a new 1.5 mL microcentrifuge tube to reduce non-specific binding. To elute proteins, the washed beads were resuspended in elution buffer containing protease inhibitor and proteinase K, incubated at 50 °C for 45 min, and then heated at 95 °C for 10 min to inactivate proteinase K. After centrifugation, the supernatant was collected as the protein solution. The resulting protein samples were separated by polyacrylamide gel electrophoresis, and differential protein bands were excised and sent to the State Key Laboratory of Subtropical Region of Guangxi University for mass spectrometry analysis.

### 2.5 Cell culture

AML12 cells were grown in DMEM (Gibco®; Thermo Fisher Scientific, China) enriched with 10% fetal bovine serum. Additionally, 1% penicillin–streptomycin was included to prevent microbial contamination. The cells were cultured at 37 °C and 5% CO_2_. Induction of adipogenesis was performed using OA/PA medium, which consists of DMEM supplemented with 1% oleic acid, 1% palmitic acid and 2% BSA. The overexpression vector for *lnc-CidecAS* was constructed by cloning the *lnc-CidecAS* sequence into the plasmid pcDNA3.1(−) via digestion and ligation. The plasmid was amplified in *Escherichia coli* cells and purified using a plasmid extraction kit (#DP117; Tiangen Biotech (Beijing) Co., Ltd., Beijing, China) to remove endotoxins. Cells were seeded 2 days prior to the experiment. Upon reaching approximately 80% confluency, cells were switched to serum-free DMEM, followed by a 12-h incubation period. Afterwards, all cells were transfected with nucleic acid-liposome complexes in Opti-MEM (Gibco®; from the same supplier) for 6-h using Hieff Trans® LipoBooster 3,000 Transfection Reagent (Cat. No. 40801ES03; Yeasen Biotechnology (Shanghai) Co., Ltd., Shanghai, China). Post-transfection, the medium was exchanged for OA/PA medium to initiate adipogenic induction. After 24 h, the culture supernatant was harvested to quantify extracellular TG content. After a gentle wash with PBS buffer, the cells were separated into two groups: the first cell sample was lysed in 200 μL of PBS using an ultrasonic disruptor (#Q800R; Qsonica LLC, Newtown, CT, United States) for enzymatic activity assays. The second group of cells was incubated at 0 ~ 4 °C for 40 min with RIPA lysis buffer (Solarbio Science and Technology Co., Ltd., Beijing, China) for cell lysis. Subsequently, the mixture was subjected to centrifugation ( 7,500×g , 10 min) for supernatant isolation. The resulting fraction was carefully aliquoted into two clean Eppendorf tubes for further analysis.

### 2.6 Western blot analysis

Total protein extraction was preceded by homogenizing tissues and cells in RIPA lysis buffer (add 1 mM PMSF; Solarbio, from the same supplier). Protein concentration was determined with a bicinchoninic acid kit (#P0012; Beyotime Biotechnology). Then samples were electrophoresed and then blotted onto PVDF membranes (Millipore, Guangzhou, China). The membranes were then blocked for 60 min using 5% non-fat milk prepared in 1 × TBST buffer. Subsequently, they were incubated with primary antibodies (Flag mouse monoclonal antibody [#14793; dilution, 1:2000; Cell Signaling Technology] and β-tubulin mouse monoclonal antibody [#AF2839; dilution, 1:1000; Beyotime Biotechnology]) at 4 °C for 16-h. An HRP-conjugated goat anti-mouse IgG antibody (#A0216; dilution, 1:1000; Beyotime Biotechnology) was then used for probing.

### 2.7 Biochemical parameter determination

The physiological indicators in this study (TGs, total cholesterol, VLDLs, and adenosine triphosphatase) were quantified using commercially available assay kits (NJJCBIO, China). When analyzing the results, protein concentrations measured with the BCA assay were adopted for normalization.

### 2.8 Analysis of RNA structure and interaction propensity

The RNAfold web server provides reliable prediction of RNA secondary structures based on the minimum free energy (MFE) method (42, 43), and tertiary structures were modeled using the 3dRNA platform from the Xiao Lab (44, 45). Three-dimensional structural models were generated and molecular docking simulations were carried out in Discovery Studio 2020 (Biovia Corp, Vélizy-Villacoublay Cedex, France). RNA-protein interaction propensities were estimated via the catRAPID omics v2 server platform^1^. The sequence information for *lnc-CidecAS* is included in Supplementary material 2.

### 2.9 RNA-seq analysis

Total RNA was extracted from mouse liver tissues using TRIzol reagent (#15596-026CN; Tsingke Biotech Co., Ltd., Beijing, China) and treated with DNase I (#D7076; Beyotime Biotechnology, Shanghai, China) to remove genomic DNA. RNA integrity was assessed using an Agilent 2100 Bioanalyzer (Agilent Technologies, Santa Clara, CA, United States), with RIN values ≥7.0. Strand-specific RNA-seq libraries were constructed using the NEBNext® Ultra™ RNA Library Prep Kit for Illumina® (Catalog # E7530, New England Biolabs, Ipswich, MA, United States), and the libraries were sequenced on an Illumina NovaSeq 6,000 platform with paired-end 150 bp reads. Raw sequencing data were quality-checked using FastQC and trimmed with Trimmomatic (v0.39) to remove low-quality bases and adapter sequences. The filtered high-quality reads were aligned to the mouse reference genome (mm10) using HISAT2 (v2.2.1). Transcript assembly and quantification were performed using StringTie (v2.1.7), and expression levels were measured as FPKM (Fragments Per Kilobase of transcript per million mapped reads). Differential expression analysis was conducted using the R package DESeq2 (v1.34.0), with genes meeting the criteria of |log_2_ fold change| ≥ 1 and adjusted *p*-value (*p*-adj) < 0.05 considered significantly differentially expressed. Volcano plots were generated using the R package ggplot2 (v3.3.5). Candidate transcripts were evaluated using four coding potential prediction tools (CPC2 (beta), CNCI (v1.0), CPAT (v1.2.1), Pfam (v33.1)), and only those consistently predicted as non-coding by all four tools were retained as lncRNAs for further analysis. The FASTA file and visualization data for *lnc-CidecAS* are available upon request from the corresponding author.

### 2.10 Statistical analysis

Data were visualized with GraphPad Prism 10 (GraphPad Software, LLC, San Diego, CA, United States). All experiments contained a minimum of three biological replicates. Results are expressed as mean ± SEM. Differences between two groups were assessed by unpaired two-tailed Student’s *t*-test, and comparisons among multiple groups were evaluated using one-way ANOVA. A *p*-value <0.05 (*) was taken to indicate statistical significance, whereas *p*-value <0.01 (**) and *p*-value <0.001 (***) were deemed highly significant. Non-significant (ns) denotes *p* ≥ 0.05.

## 3 Results

### 3.1 Identification and expression pattern of *lnc-CidecAS*

**Figure 1:**
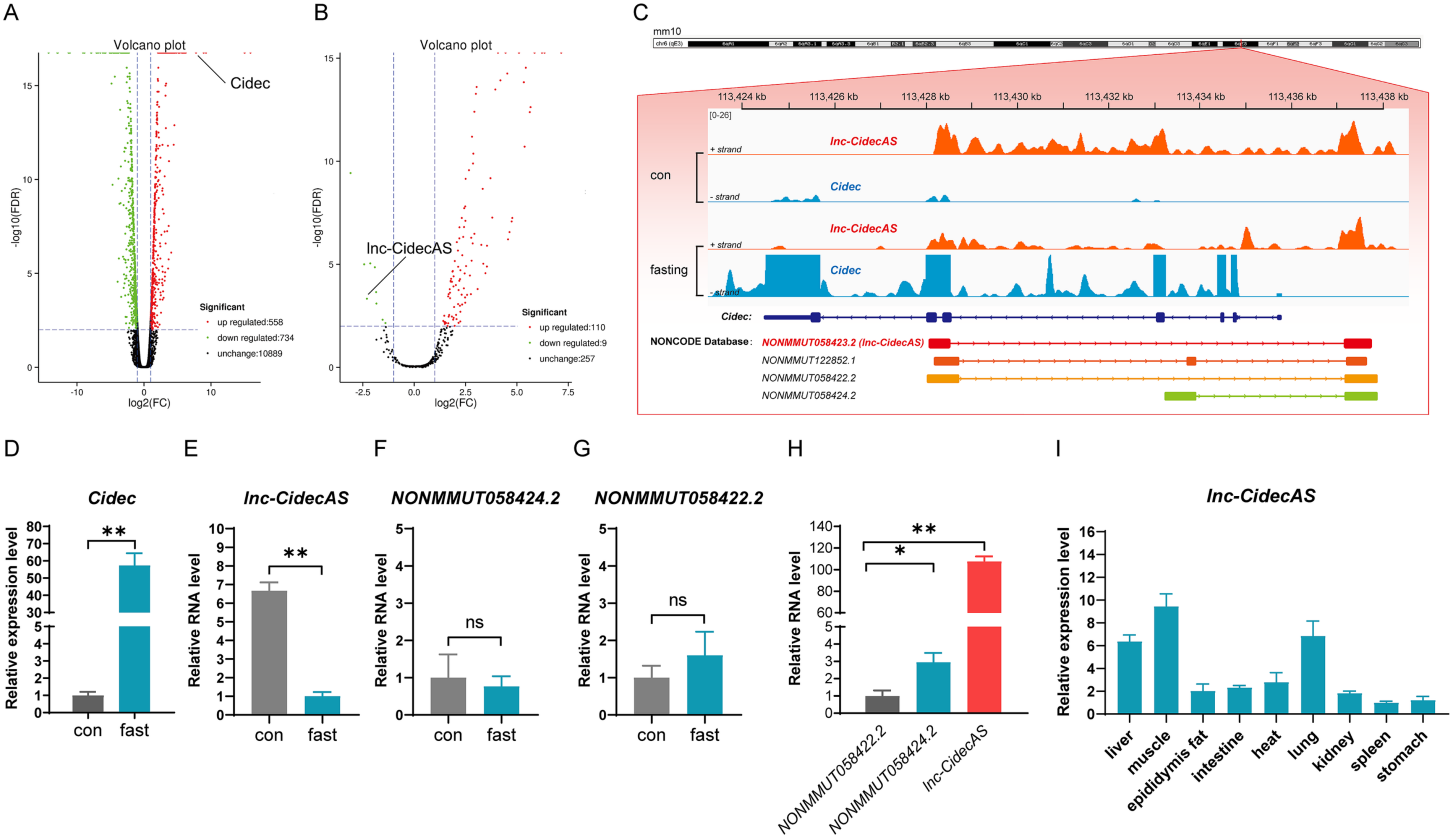
Characterization of *lnc-CidecAS*. **(A,B)** Volcano plot showing differentially expressed genes and lncRNAs between control and fasted mice liver. **(C)** Relative genomic positions of the *Cidec* gene, *lnc-CidecAS* and several subtypes in mice. **(D–G)** Changes in expression levels of the *Cidec* (*n* = 5), *lnc-CidecAS* and two subtypes in mouse liver after fasting (*n* = 3). **(H)** Relative expression levels of *lnc-CidecAS* and its two detectable subtypes in the normal liver (*n* = 3). **(I)** Expression distribution of *lnc-CidecAS* in various tissues of mice (*n* = 4). Data were normalized to the housekeeping gene and expression values were calculated relative to the control group mean.

*Cidec* is a key gene regulating lipid droplet fusion. In our previous study investigating the effects of fasting on *Cidec* expression levels (Figure 1A) (46), we identified a differentially expressed novel lncRNA on the antisense strand by lncRNA sequencing (Figure 1B). This lncRNA is located on the antisense strand of the *Cidec* gene in the mouse chromosome 6 (qE3) region; therefore, it was temporarily designated as *lnc-CidecAS*. Based on its genomic location and partial sequence information, a search in the NONCODE database^2^ revealed its accession number as NONMMUT058423.2, and three additional lncRNA subtypes (NONMMUT122852.1, NONMMUT058422.2, and NONMMUT058424.2) were also identified at this locus. The positional relationships among *Cidec*, *lnc-CidecAS*, and related subtypes are illustrated in Figure 1C. Subsequently, specific quantitative primers for these transcripts were designed for qPCR analysis. The results showed that *Cidec* expression was increased by more than 50-fold under fasting conditions (Figure 1D). In the mouse liver, expression of *lnc-CidecAS* was reduced to approximately 1/6 of that in the normal diet group (Figure 1E). Moreover, the expression levels of the other subtypes of *lnc-CidecAS* in the liver did not exhibit significant changes (Figures 1F,G). Among these, *NONMMUT122852.1* was undetectable due to low expression. Notably, *lnc-CidecAS* showed the lowest Ct value among the detectable transcripts under the same experimental conditions (Figure 1H), and its expression was relatively higher in liver, muscle, and lung tissues (Figure 1I). Therefore, it was selected as the focus of this study.

**Figure 2:**
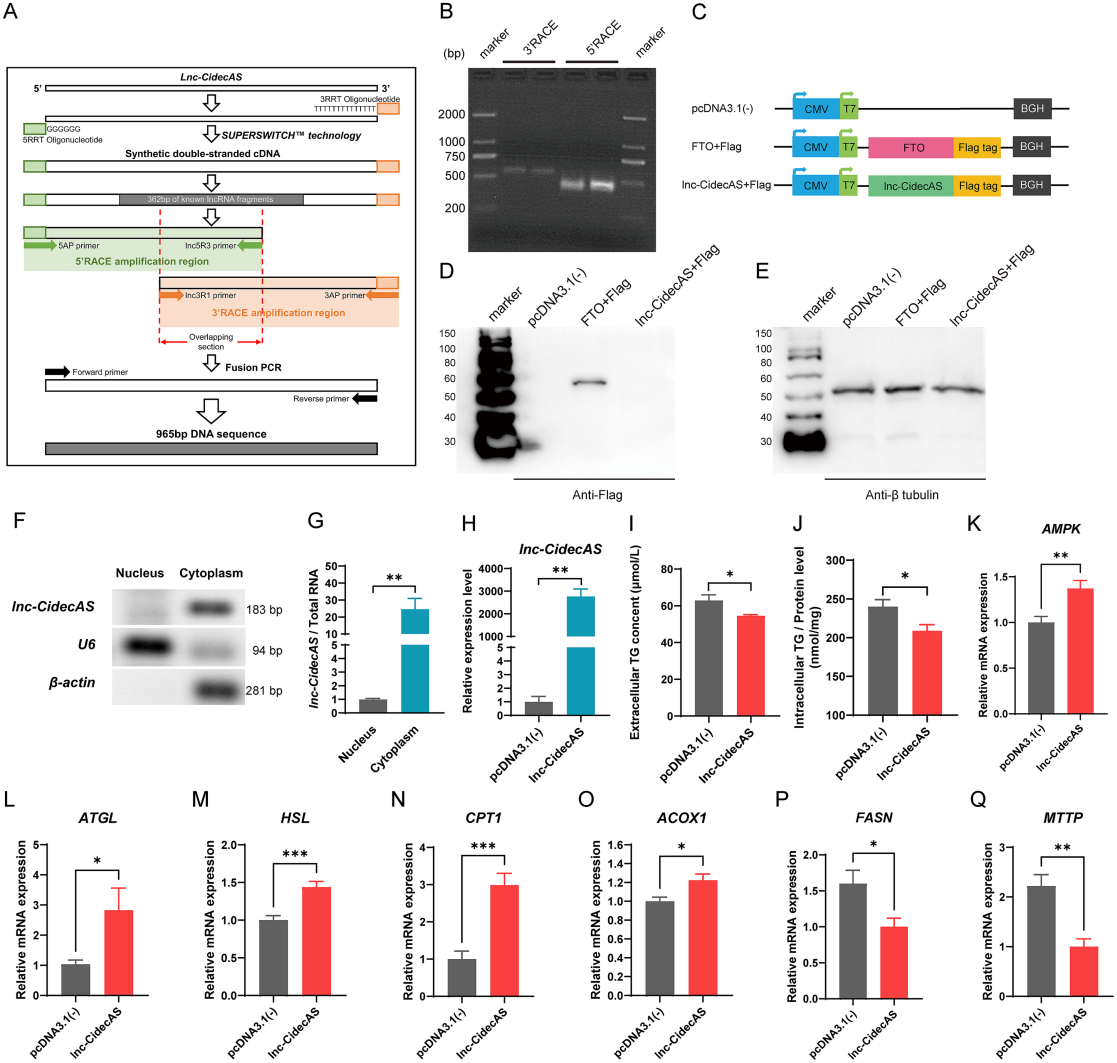
Sequence structure and coding capacity confirmation of *lnc-CidecAS*. **(A)** Schematic diagram of the workflow for 5′ and 3′ RACE targeting *lnc-CidecAS*. **(B)** Agarose gel electrophoresis of two sequence fragments obtained from RACE experiments, excluding overlapping 12 bp and respective adapter primers: the 3′ fragment is 501 bp, and the 5′ fragment is 476 bp. **(C)** Schematic diagram of key structures for the PCDNA3.1(−), FTO + flag, and *lnc-CidecAS* + flag plasmids. **(D–E)** Western blotting results showing the ability of three groups of cells to express flag-tagged proteins. PCDNA3.1(−) serves as a negative control, FTO + flag acts as the positive control, and *β*-tubulin is used as a background control. **(F,G)** RT-PCR analysis of RNA purified from cytoplasmic and nuclear fractions of mouse hepatocytes, followed by gel electrophoresis to analyze relative content. Images were inverted and quantified. Among them, *U6* serves as a nuclear marker, and β-actin serves as a cytoplasmic marker (*n* = 4). **(F)** RT-PCR analysis of subcellular fractions. Total RNA was extracted from nuclear and cytoplasmic fractions isolated from mouse liver. RT-PCR was performed using U6 (nuclear marker) and β-actin (cytoplasmic marker), and the products were analyzed by gel electrophoresis to detect the band distribution of *lnc-CidecAS*. Images were inverted to enhance band visibility. **(G)** qPCR analysis of subcellular fractions. Relative expression levels were calculated using the 2^−ΔΔCT^ method and normalized to the total RNA concentration of the nuclear and cytoplasmic fractions, respectively. Results showed that *lnc-CidecAS* is predominantly enriched in the cytoplasm (*n* = 4). **(H–J)** *lnc-CidecAS* expression levels (*n* = 4), extracellular and intracellular TG concent after transfection with the *lnc-CidecAS* expression vector in AML12 cells (*n* = 6). **(K–Q)** Changes in gene expression levels of lipid metabolism-related genes in AML12 cells after overexpression of *lnc-CidecAS* (*n* ≥ 5).

Based on the lncRNA sequencing predictions, the 5′ and 3′ RACE techniques were employed to amplify the ends of *lnc-CidecAS* (Figure 2A), ultimately obtaining a complete sequence of 965 base pairs (Figure 2B). To verify whether *lnc-CidecAS* possesses protein-coding capabilities, the sequence was inserted into the pcDNA3.1(−) plasmid containing cytomegalovirus and T7 promoters, and a Flag tag appended at the 3′ end (Figure 2C), which was then transfected into mouse AML12 cells for expression. The results demonstrated that *lnc-CidecAS* lacks protein-coding ability (Figures 2D,E), which is largely consistent with the protein-coding capability prediction websites (Table 1). Isolation of the nuclei and cytoplasm of mouse liver cells and subsequent RNA extraction found that *lnc-CidecAS* is primarily enriched in the cytoplasm (Figures 2F,G), aligning with the predictions from the cited websites (Table 2).

**Table 1:** The CPC tool predicted result.

Name	Coding/noncoding	Coding potential score	Frame score	FrameFinder LOG-ODDS score
*lnc-CidecAS*	Noncoding (weak)	−0.91	0.0	37.61
*LncMyoD*	Noncoding (weak)	−0.77	0.0	42.38
*FTO*	Coding	9.88	6107.26	232.73

LncMyoD as a representative of lncRNA, and FTO as a representative of protein-coding gene. The website address is http://cpc.cbi.pku.edu.cn/.

**Table 2:** lncLocator tool prediction result.

Subcellular locations	Score
Cytoplasm	0.68
Nucleus	0.23
Ribosome	0.02
Cytosol	0.03
Exosome	0.04
Prediction location	Cytoplasm

The website address is http://www.csbio.sjtu.edu.cn/bioinf/lncLocator/index.html.

### 3.2 *lnc-CidecAS* affects lipid metabolic processes in hepatocytes

A eukaryotic expression vector carrying *lnc-CidecAS* was constructed and successfully transfected into mouse AML12 cells to elucidate the function of *lnc-CidecAS* in the regulation of lipid metabolism in hepatocytes (Figure 2H). The data showed that *lnc-CidecAS* significantly reduced TG levels in the extracellular medium and in the intracellular fraction (Figures 2I,J), while concurrently increasing the gene expression levels of *AMPK*, *ATGL*, *HSL*, *CPT1*, *ACOX1*, *FASN*, and *MTTP* (Figure 2K–Q). In contrast, the expression levels of *PFK*, *CIDEC*, *PPARG*, *SREBP1*, *ACC* and *ACC2* remained largely unchanged upon *lnc-CidecAS* overexpression (Supplementary Figures 1A–D).

### 3.3 *lnc-CidecAS* reduced body fat accumulation in normal mice

**Figure 3:**
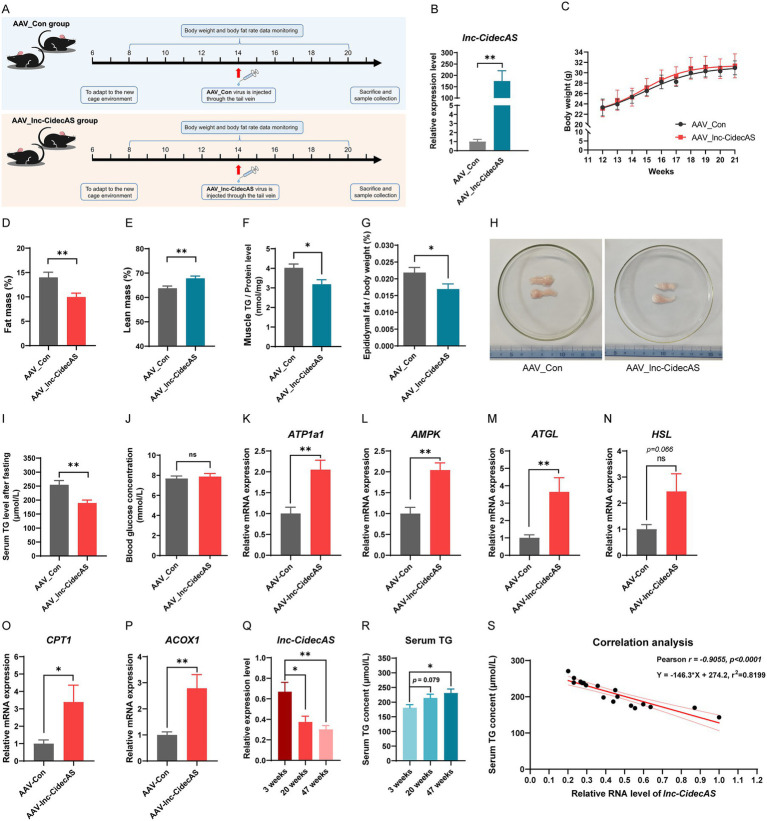
Effects of overexpressing *lnc-CidecAS* on mouse physiological functions under fasting conditions. **(A)** Experimental workflow diagram for liver-specific infection of AAV_*lnc-CidecAS* virus in C57BL/6J mice (*n* = 6). **(B)** Expression levels of *lnc-CidecAS* in the livers of two groups of mice after the experiment (*n* = 4). **(C–H)** Weight gain curves, fat mass percentage, lean mass percentage, intramuscular fat content, relative weight of epididymal fat, and epididymal fat tissue for two groups of mice (*n* = 6). **(I,J)** Serum TG and blood glucose levels after 12 h of fasting in both groups of mice (*n* = 6). **(K–P)** mRNA levels of *ATP1a1*, *AMPK*, *ATGL*, *HSL*, *CPT1*, and *ACOX1* in the liver of normal diet fed mice determined by qPCR (*n* = 6). **(Q,R)** Expression levels of *lnc-CidecAS* in the liver and serum TG levels in wild-type mice of different ages (*n* = 6). **(S)** Analysis of the correlation between *lnc-CidecAS* expression in the liver and serum TG levels in mice at 3, 20, and 47 weeks of age (*n* = 18).

In the fasting state, *lnc-CidecAS* expression was significantly reduced (Figure 1E), while overexpression in cells significantly decreased TG levels in the extracellular medium (Figure 2I), suggesting that *lnc-CidecAS* might be involved in lipid metabolism of liver cells. To test this hypothesis, normal mice were administered a tail vein injection of AAV-8 carrying *lnc-CidecAS* for overexpression in the liver (Figure 3A). As shown in Figure 3B, AAV_*lnc-CidecAS* was successfully overexpressed in the liver and body weight gain did not differ significantly between the two groups (Figure 3C). Whereas at week 21, the *lnc-CidecAS* group demonstrated a marked decrease in body fat percentage relative to the control group (Figure 3D), with a marked increase in lean body mass (Figure 3E), while the intramuscular fat content was significantly lower (Figure 3F). Additional investigation showed that epididymal fat content was markedly reduced in the *lnc-CidecAS* group (Figures 3G,H). Furthermore, after 24 h of fasting, serum TG levels were also decreased (Figure 3I). Notably, there was no effect on blood glucose levels (Figure 3J). In normal diet-fed mice, overexpression of *lnc-CidecAS* significantly increased the mRNA levels of *ATP1a1*, *AMPK*, *ATGL*, *CPT1*, and *ACOX1* in the liver, while *HSL* expression also showed an upward trend without reaching statistical significance (Figures 3K–P). These results are highly consistent with those from the cellular overexpression experiments (Figures 2K–O). Additionally, liver expression of *lnc-CidecAS* decreased significantly with age in wild-type mice at ages 3, 20 and 47 weeks (Figure 3Q), while serum TG levels progressively increased (Figure 3R), exhibiting a strong negative correlation between the two variables (*r* = −0.9055, *r*^2^ = 0.8199, Figure 3S).

### 3.4 *lnc-CidecAS* significantly improved the blood lipid profile of mice under high-fat diet conditions

**Figure 4:**
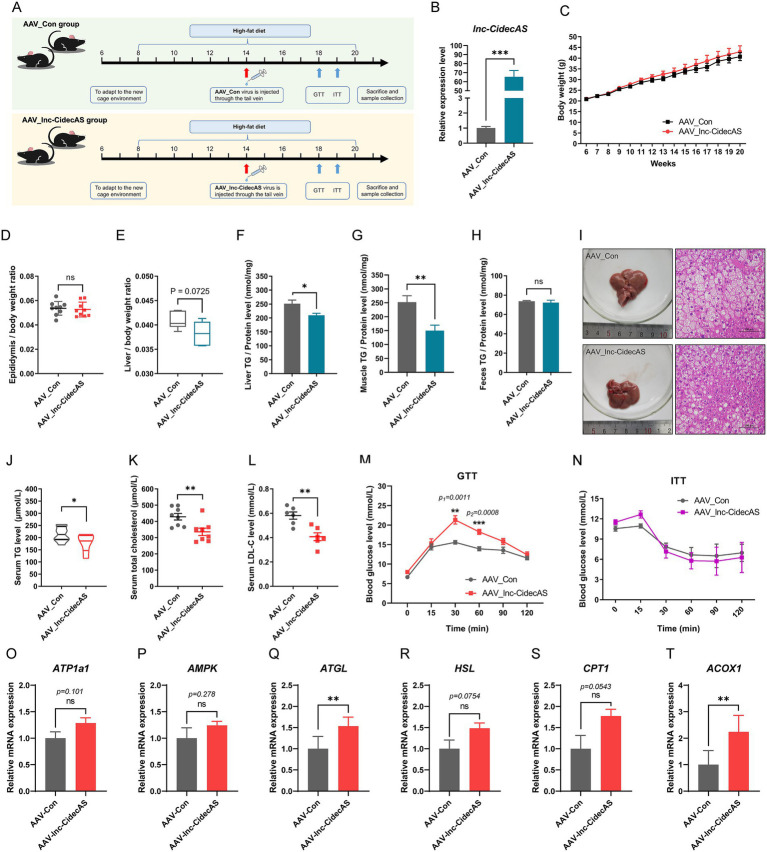
Effects of overexpressing *lnc-CidecAS* on mouse physiological functions under a high-fat diet. **(A)** Experimental workflow diagram for liver-specific infection of AAV_*lnc-CidecAS* virus in C57BL/6J mice under a high-fat diet (*n* = 6). **(B)** Expression levels of *lnc-CidecAS* in the livers of two groups of mice after the experiment (*n* = 6). **(C–H)** Weight gain curves, relative weight of epididymal fat, relative liver weight, liver TG levels, intramuscular fat content, and fecal TG content for two groups of mice under a high-fat diet (*n* = 6). **(I)** Photographs and H&E-stained sections of two groups mouse livers. **(J–L)** Serum TG, total serum cholesterol, and LDL-C levels in both groups of mice (*n* = 6). **(M,N)** GTT and ITT curves measured after 18 weeks for both groups of mice (*n* = 5). **(O–T)** mRNA levels of *ATP1a1*, *AMPK*, *ATGL*, *HSL*, *CPT1*, and *ACOX1* in the liver of HFD-fed mice determined by qPCR (*n* = 6).

Given that *lnc-CidecAS* can influence body fat accumulation and blood lipid levels in fasting mice, we wondered whether there would be a similar effect under high-fat diet conditions. Notably, compared with normal chow feeding, high-fat diet significantly reduced the expression of *lnc-CidecAS* in mouse liver (to approximately one-seventh; Supplementary Figure 2A). Therefore, we overexpressed *lnc-CidecAS* in the livers of HFD-fed mice (Figure 4A), which was achieved in both groups (Figure 4B). During the entire experiment, two groups showed comparable rates of body weight gain (Figure 4C) and no significant difference in the AST/ALT ratio (Supplementary Figure 2B). However, under high-fat diet conditions, overexpression of *lnc-CidecAS* did not lead to a reduction in epididymal fat weight (Figure 4D), but did decrease the liver weight (Figure 4E). Further analysis revealed that the *lnc-CidecAS* group had decreased TG levels in the liver (Figure 4F) and reduced intramuscular fat content (Figure 4G), but no difference in fecal TG levels (Figure 4H). Hepatic steatosis was assessed by Oil Red O staining, which revealed decreased fat deposition in the *lnc-CidecAS* group (Figure 4I), whereas overexpression of *lnc-CidecAS* resulted in significant reductions in serum TGs, total cholesterol, and LDL-C levels (Figures 4J–L). Data from the glucose and insulin tolerance test demonstrated that overexpression of *lnc-CidecAS* weakened glucose tolerance in mice (Figure 4M), with no significant effect on insulin tolerance (Figure 4N). Under high-fat diet conditions, overexpression of *lnc-CidecAS* led to upward trends in the mRNA levels of *ATP1a1*, *AMPK*, *HSL*, and *CPT1* in the liver without reaching statistical significance, whereas the expression of *ATGL* and *ACOX1* was significantly increased (Figures 4O–T). These results are generally consistent with those observed in cellular experiments and in normal diet-fed mice, although the effects appeared to be attenuated under high-fat diet conditions, suggesting that the metabolic stress induced by HFD may partially counteract the regulatory role of *lnc-CidecAS*.

### 3.5 *lnc-CidecAS* influenced lipid metabolism regulation via ATP1a1

**Figure 5:**
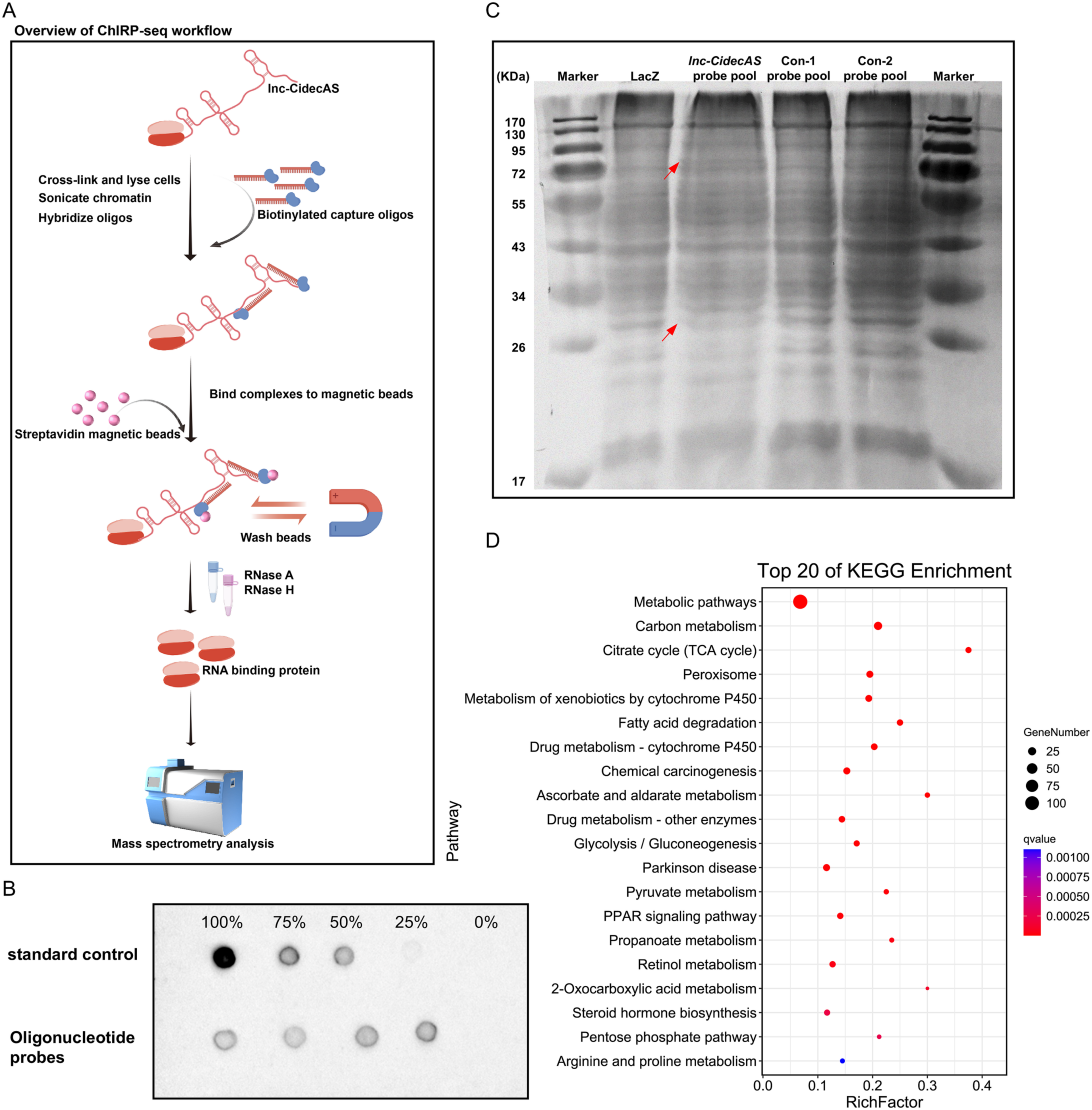
Target protein acquisition of *lnc-CidecAS*. **(A)** Overview of ChIRP-seq workflow. **(B)** Efficiency determination of biotin labeling for partial oligonucleotide probes. **(C)** Differential bands specifically bound to *lnc-CidecAS* through the ChRIP experiment; nucleic acid-protein complexes were eluted and separated by SDS-PAGE, followed by Coomassie Brilliant Blue staining (grayscale processed). **(D)** After mass spectrometry analysis, KEGG enrichment analysis was performed on proteins. The top 20 signaling pathways from the KEGG enrichment analysis.

Given that *lnc-CidecAS* affects lipid metabolism in the liver and blood, we were intrigued by the mechanism of action. Hence, 10 specific oligonucleotide probes targeting *lnc-CidecAS* were designed and labeled with biotin. Then, ChIRP technology was used to identify potential targets of *lnc-CidecAS* (Figures 5A,B). The lysates from mouse liver cells were co-incubated with these probes, followed by multiple rounds of magnetic bead adsorption, washing, and separation, which led to the capture of distinct bands representing *lnc-CidecAS*-bound complexes (Figure 5C). The captured proteins were identified by mass spectrometry (Supplementary material 3). Their top 20 signaling pathways were analyzed by Kyoto Encyclopedia of Genes and Genomes (KEGG) enrichment. This analysis revealed their involvement in various metabolic processes, including carbon metabolism, the citric acid cycle, peroxisome, fatty acid degradation, glycolysis/gluconeogenesis, pyruvate metabolism, PPAR signaling pathway, propanoate metabolism and steroid hormone biosynthesis (Figure 5D). KEGG enrichment analysis also identified proteins associated with lipid metabolism pathways. Subsequent prediction of RNA-protein interactions tendencies with the catRAPID omics v2 server identified ATP1a1 as a high-scoring protein with strong binding potential (Table 3, Supplementary material 4).

**Table 3:** RNA-protein interaction tendencies prediction.

Protein_ID	RNA_ID	Interaction_propensity	*Z*_score	RBP_propensity	Ranking
**sp.Q8VDN2.AT1A1 (ATP1a1)**	**lnc-CidecAS**	**0.63**	**3.14**	**1**	**71.14**
tr.Q3T9X3.Q3T9X3	*lnc-CidecAS*	0.61	2.53	1	60.47
tr.Q3UEJ6.Q3UEJ6	*lnc-CidecAS*	0.42	3.53	0.33	78.01
sp.P70227.ITPR3	*lnc-CidecAS*	0.38	2.72	0.29	63.68
tr.A0A286YCI8.A0A286YCI8	*lnc-CidecAS*	0.38	1.95	0.39	50.28
tr.B7ZC18.B7ZC18	*lnc-CidecAS*	0.37	1.9	0.36	49.34
sp.E9Q4Z2.ACACB	*lnc-CidecAS*	0.36	1.95	0.34	50.18
sp.Q64521.GPDM	*lnc-CidecAS*	0.36	1.82	0.36	48.04
sp.P12382.PFKAL	*lnc-CidecAS*	0.34	2.48	0.21	59.45
tr.Q3UEJ6.Q3UEJ6	*lnc-CidecAS*	0.34	1.58	0.33	43.77

The website address is http://service.tartaglialab.com/page/catrapid_omics2_group.

**Figure 6:**
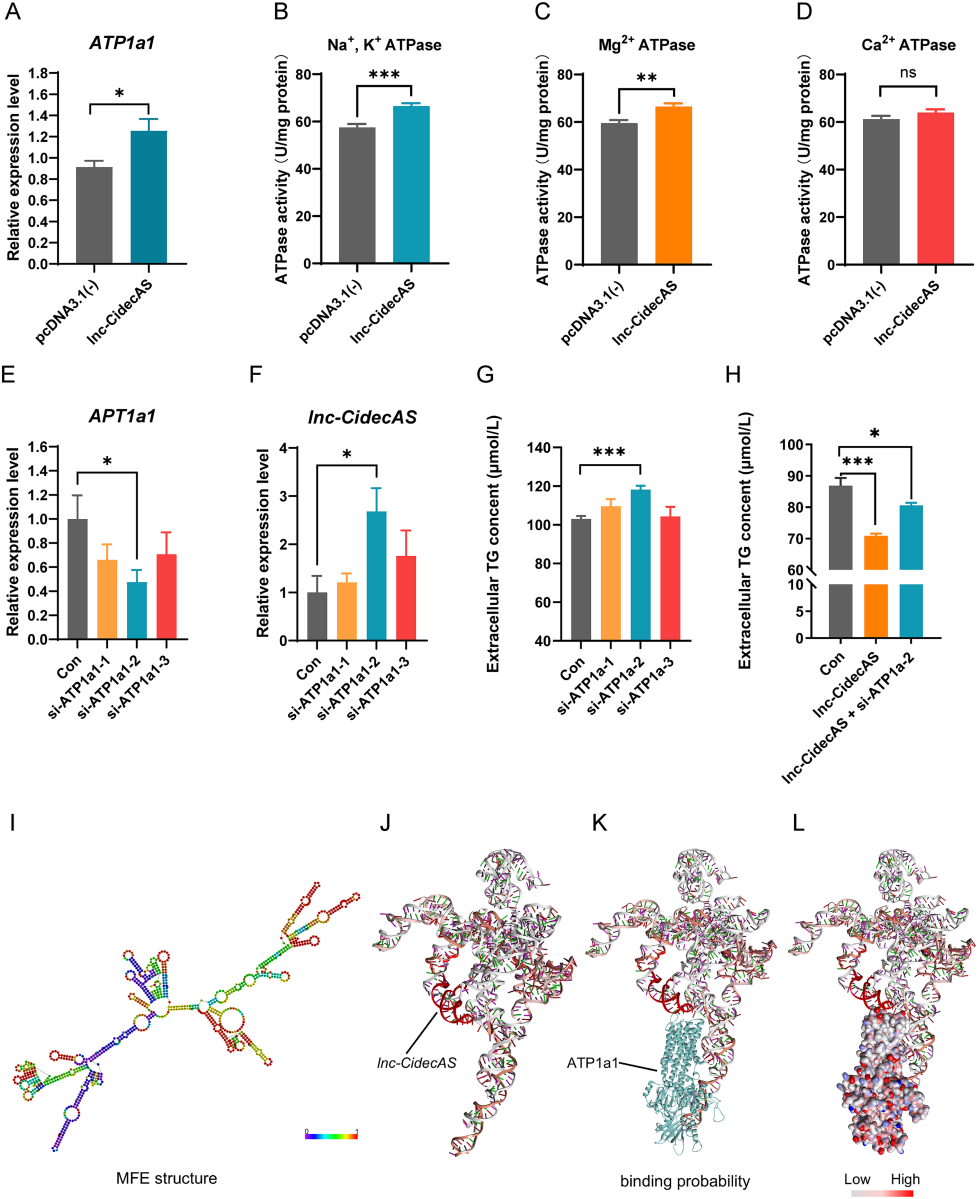
*lnc-CidecAS* binds to ATP1a1 and jointly regulates lipid metabolism in hepatocytes. **(A–D)** Changes in *ATP1a1* gene expression levels and enzyme activity assays in AML12 cells after overexpression of *lnc-CidecAS* (*n* = 6). **(E,F)** Expression levels of *ATP1a1* and *lnc-CidecAS* after *ATP1a1* gene knockdown using siRNA in AML12 cells (*n* = 3). **(G)** Extracellular TG levels after *ATP1a1* knockdown in AML12 cells (*n* = 6). **(H)** Rescue experiment in AML12 cells, where *lnc-CidecAS* is overexpressed while *ATP1a1* is knocked down, observing changes in extracellular TG levels (*n* = 8). **(I)** Secondary structure diagram of *lnc-CidecAS* predicted based on the minimum free energy (MFE) method. **(J–L)** Predicted tertiary structure diagram of *lnc-CidecAS* along with its potential binding sites with ATP1a1. The darker the red color in the segments, the higher the potential of binding.

To verify whether ATP1a1 functions as a regulatory target of *lnc-CidecAS* in lipid metabolism, the effect of *lnc-CidecAS* overexpression on ATP1a1 expression in AML12 liver cells was investigated. The results demonstrated that ATP1a1 expression increased as *lnc-CidecAS* was overexpressed (Figure 6A). Moreover, cellular enzyme activity assays demonstrated that both sodium-potassium ATPase and magnesium ATPase activities significantly increased with overexpression of *lnc-CidecAS* (Figures 6B,C), whereas calcium ATPase activity remained unchanged (Figure 6D). Subsequently, we performed knockdown experiments targeting the ATP1a1 gene and selected si-ATP1a1-2, which exhibited the highest knockdown efficiency, for further experiments (Figure 6E). Following ATP1a1 knockdown, there was a significant increase in *lnc-CidecAS* expression levels (Figure 6F), accompanied by a marked elevation in extracellular TG levels (Figure 6G). Rescue experiments showed that co-transfection with si-ATP1a1-2 partially reversed the decreasing trend of extracellular TG levels in the presence of *lnc-CidecAS* overexpression (Figure 6H). Based on the minimum free energy method, we predicted the secondary structure (Figure 6I) and tertiary structure (Figure 6J) of *lnc-CidecAS*, which revealed the presence of multiple stem-loop structures. Molecular docking simulations identified several binding domains between *lnc-CidecAS* and ATP1a1 (Figures 6K,L).

## 4 Discussion

Previous studies have identified *Cidec* as a key gene located at the contact sites of lipid droplets, playing an essential role in lipid droplet fusion and lipid storage regulation (47–49). Specifically, targeted knockout of this gene disrupts lipid metabolism in the intestine, leading to reduced lipid intake and accumulation (46). In this study, the novel lncRNA (*lnc-CidecAS*) located on the antisense strand of the *Cidec* gene was identified and the expression patterns as well as physiological functions were investigated under different nutritional states. Additionally, target proteins were preliminarily characterized.

Based on the relative positioning of lncRNAs within genes, *lnc-CidecAS* is classified as a classical antisense lncRNA (50, 51). In fact, it is more common for antisense lncRNAs to directly regulate nearby genes. For example, *Gm15441*, an antisense lncRNA located on the antisense strand, regulates lipid metabolism by controlling expression of the *Txnip* gene (52). Similarly, the antisense lncRNA *Hnf4αos* stabilizes *Hnf4α* mRNA through generating a dsRNA complex, which activates miR-23a, leading to inhibition of PGC1α expression and exacerbating liver ischemia–reperfusion injury in mice (53). While we currently lack direct evidence that *lnc-CidecAS* is the key molecule mediating the role of Cidec in lipid regulation, ChIRP combined with mass spectrometry demonstrated that *lnc-CidecAS* can interact with ATP1a1 to enhance lipid oxidation, metabolism, and transport. This interaction reduces lipid accumulation in extracellular compartments and serum, ultimately influencing systemic lipid metabolism. This mechanism is similar to that of the lncRNA *ADIPINT*, which directly binds and regulates pyruvate carboxylase to modulate lipid regulation (54). In both cases, the effects of lncRNAs are exerted by directly binding transcription factors (55, 56), RNA-binding proteins (57, 58), or metabolic enzymes (54, 59) within pathways related to fat metabolism or inflammation.

This study identifies *lnc-CidecAS* as a potent regulator of hepatic lipid metabolism and suggests the existence of a functional axis involving ATP1a1. At the proximal node of this putative axis, *lnc-CidecAS* directly binds to and stabilizes ATP1a1, substantially upregulating its mRNA expression and enzymatic activity, while ATP1a1 in turn exerts a negative feedback on *lnc-CidecAS* expression, forming a precisely controlled regulatory unit. The enhanced ATP1a1 activity subsequently activates the cellular energy sensor *AMPK*, which serves as a central hub transducing upstream signals to downstream metabolic effectors. Activated *AMPK* orchestrates a bidirectional metabolic reprogramming: on one hand, it promotes lipolysis via upregulation of *ATGL* and *HSL*, and unleashes fatty acid oxidation by relieving *CPT1* inhibition through *ACC* inhibition (despite unchanged *ACC* mRNA), synergizing with *ACOX1* upregulation to drive mitochondrial and peroxisomal β-oxidation, thereby depleting intracellular lipid stores; on the other hand, it directly suppresses *FASN* to curtail *de novo* lipogenesis and robustly inhibits *MTTP*, the rate-limiting component of VLDL assembly, effectively blocking hepatic lipid export. This dual action collectively accounts for the reduction in intracellular triglycerides (primarily driven by enhanced catabolism) and extracellular/serum triglycerides (primarily attributable to suppressed secretion), culminating in ameliorated hepatic steatosis and improved serum lipid profiles *in vivo*.

Supporting evidence further defines the specificity of this regulatory circuit. First, although *lnc-CidecAS* genomically overlaps with the *Cidec* gene, its overexpression did not alter *Cidec* mRNA levels, confirming that *lnc-CidecAS* functions as an independent trans-acting factor rather than a cis-regulator of its neighboring gene. This distinction excludes positional effect confounders and underscores the novelty of *lnc-CidecAS* as a bona fide functional lncRNA. Second, the expression of master lipogenic transcription factors *PPARγ* and *SREBP1* remained unaffected, indicating that the *lnc-CidecAS*-ATP1a1 axis operates independently of global transcriptional reprogramming. Instead, it selectively modulates key metabolic enzymes-downregulating *FASN* and *MTTP* while upregulating *ATGL*, *HSL*, *CPT1*, and *ACOX1*-via specific engagement of the *AMPK* signaling pathway, thereby achieving precise metabolic flux redirection. Additionally, while *lnc-CidecAS* overexpression led to mildly impaired glucose tolerance, the expression of the glycolytic rate-limiting enzyme *PFK* was not significantly altered, suggesting that this axis exerts limited direct effects on glucose metabolism and primarily targets lipid homeostasis.

We acknowledge that the present study has not fully elucidated the direct signaling linkage between ATP1a1 and *AMPK*; the precise molecular events by which enhanced ATP1a1 activity triggers *AMPK* phosphorylation warrant further investigation. In addition, the specific binding interface between *lnc-CidecAS* and ATP1a1 has yet to be precisely mapped through structural prediction or truncation mutation scanning, and ATP1a1 protein levels in animal tissues were not examined. Addressing these gaps—particularly through structural mapping, site-directed mutagenesis, and in vivo protein validation—will be a key focus of our future studies to further consolidate this regulatory axis.

In this study, overexpression of *lnc-CidecAS* not only reduced blood TG and cholesterol levels in HFD-fed mice, but also substantially lowered LDL-C concentrations. Previous studies reported that a decrease in LDL-C levels can considerably reduce the incidence of atherosclerotic cardiovascular disease (60–62). However, the mRNA levels of some downstream targets (*ATP1a1*, *AMPK*, *HSL* and *CPT1*) showed only non-significant upward trends. This discrepancy is not contradictory. HFD-induced metabolic stress (insulin resistance, inflammation, oxidative stress) may partially attenuate transcriptional activation of these genes, while others such as *ATGL* and *ACOX1* remained significantly upregulated (Figures 4Q,T), suggesting that they are more sensitive to *lnc-CidecAS* regulation. The combined synergistic upregulation of multiple genes likely contributes to the robust lipid-lowering phenotype. Moreover, post-translational regulation (e.g., enhanced ATP1a1 enzymatic activity) may play a more direct role than mRNA changes. Additionally, *lnc-CidecAS* expression gradually decreased with the increasing age of mice, whereas serum TG levels progressively increased, exhibiting a significant negative correlation. These findings suggest that *lnc-CidecAS*, upon binding to ATP1a1, not only exerts a lipid-lowering effect but may also be crucial to maintain lipid homeostasis during the aging process. Therefore, maintaining *lnc-CidecAS* at normal levels could potentially improve vascular health. Additionally, overexpression of *lnc-CidecAS* increased ATP1a1 expression and enhanced Na^+^/K^+^-ATPase activity, while simultaneously increasing Mg^2+^-ATPase activity. Elevated Na^+^/K^+^-ATPase activity correlates with improved fatty acid metabolism and reduced oxidative damage (63, 64). Increased Mg^2+^-ATPase activity may reflect cellular adaptation to increased energy demands or magnesium ion metabolic disorders (65–67). On the other hand, unaltered Ca^2+^-ATPase activity suggests that intracellular calcium signaling pathways are not significantly affected or that calcium ion efflux mechanisms are not activated (68, 69). Considering these changes to ATPase activities, overexpression of *lnc-CidecAS* may place cells in a state of high energy demand or fatty acid oxidative stress, consistent with the previously observed upregulation of lipid oxidation-related genes, thereby necessitating increased ATPase activity to maintain ion gradients.

Subsequently, siRNAs were used to inhibit ATP1a1, which led to a compensatory increase in *lnc-CidecAS* expression and elevated extracellular TG levels. This observation suggests the existence of a negative feedback loop between ATP1a1 and *lnc-CidecAS*. Under normal conditions, *lnc-CidecAS* promotes ATP1a1 expression and enzymatic activity (Figures 3K, 6B,C). Upon *ATP1a1* knockdown, the cell attempts to compensate by upregulating *lnc-CidecAS*; however, the resulting increase in *lnc-CidecAS* fails to promote efficient TG clearance due to insufficient ATP1a1 function. Consistently, co-transfection with si-ATP1a1 partially reversed the TG-lowering effect of *lnc-CidecAS* overexpression (Figure 6H), further supporting that functional cooperation between *lnc-CidecAS* and ATP1a1 is required for lipid metabolism regulation. To date, several lncRNAs sensitive to fasting or high-fat diets have been identified, although the mechanisms of metabolic regulation significantly differ. For instance, the recently discovered *lncLMS* inhibits fat synthesis by modulating the SREBP1c pathway via feedback inhibition (70). In contrast, *lnc-CidecAS* and ATP1a1 exhibit a significant synergistic stimulation effect, jointly promoting lipid oxidation metabolism and transport. Nonetheless, our current work has not completely ruled out the possibility that *lnc-CidecAS* may also act on other targets besides ATP1a1. Future studies are expected to uncover additional proteins or nucleic acids involved in *lnc-CidecAS*-related metabolic pathways.

These findings expand the scope of research into how lncRNAs regulate cellular lipid metabolism through collaborative promotion with proteins. Mouse *lnc-CidecAS* shares a 30% sequence-conserved region with the human *CIDEC* gene (Supplementary material 5), whereas no full-length homologous transcript has been identified in humans, which is consistent with the generally low cross-species conservation of lncRNAs (71). Nevertheless, the downstream ATP1a1-AMPK signaling axis it regulates is highly conserved in mammals, and thus this study provides valuable insights into the pathogenesis of MAFLD. Based on the functional characteristics of *lnc-CidecAS* binding to ATP1a1 and regulating lipid metabolism, we speculate that targeting the *lnc-CidecAS*-ATP1a1 complex could serve as a newly developed treatment modality for combating obesity and hyperlipidemia-related conditions. This discovery provides theoretical support for the development of innovative treatment approaches for metabolic disorders.

## Data Availability

The original contributions presented in the study are included in the article/Supplementary material, further inquiries can be directed to the corresponding authors.

## Footnotes

1
http://service.tartaglialab.com/page/catrapid_omics2_group

2
https://ngdc.cncb.ac.cn/databasecommons/
