# Supplementary figures and images for: The long non-coding RNA CidecAS regulates hepatocyte lipid metabolism via the alpha-1 subunit of Na+/K+-ATPase
Source: Front Nutr. 2026 May 12;13:1809132. doi: 10.3389/fnut.2026.1809132 (PMC13201432; doi:10.3389/fnut.2026.1809132)

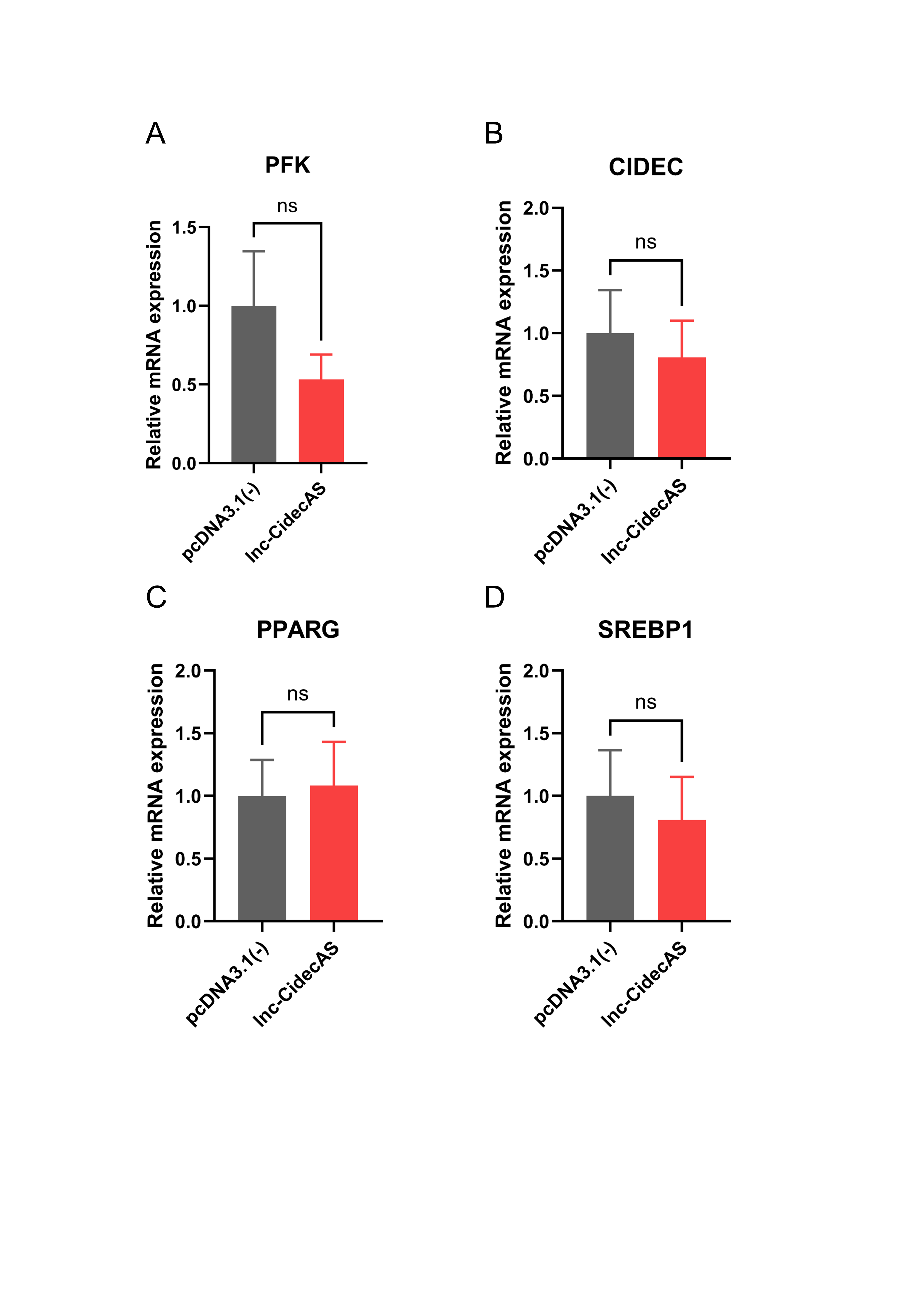

Supplement: Supplementary file 3 [file Image_1.TIF]

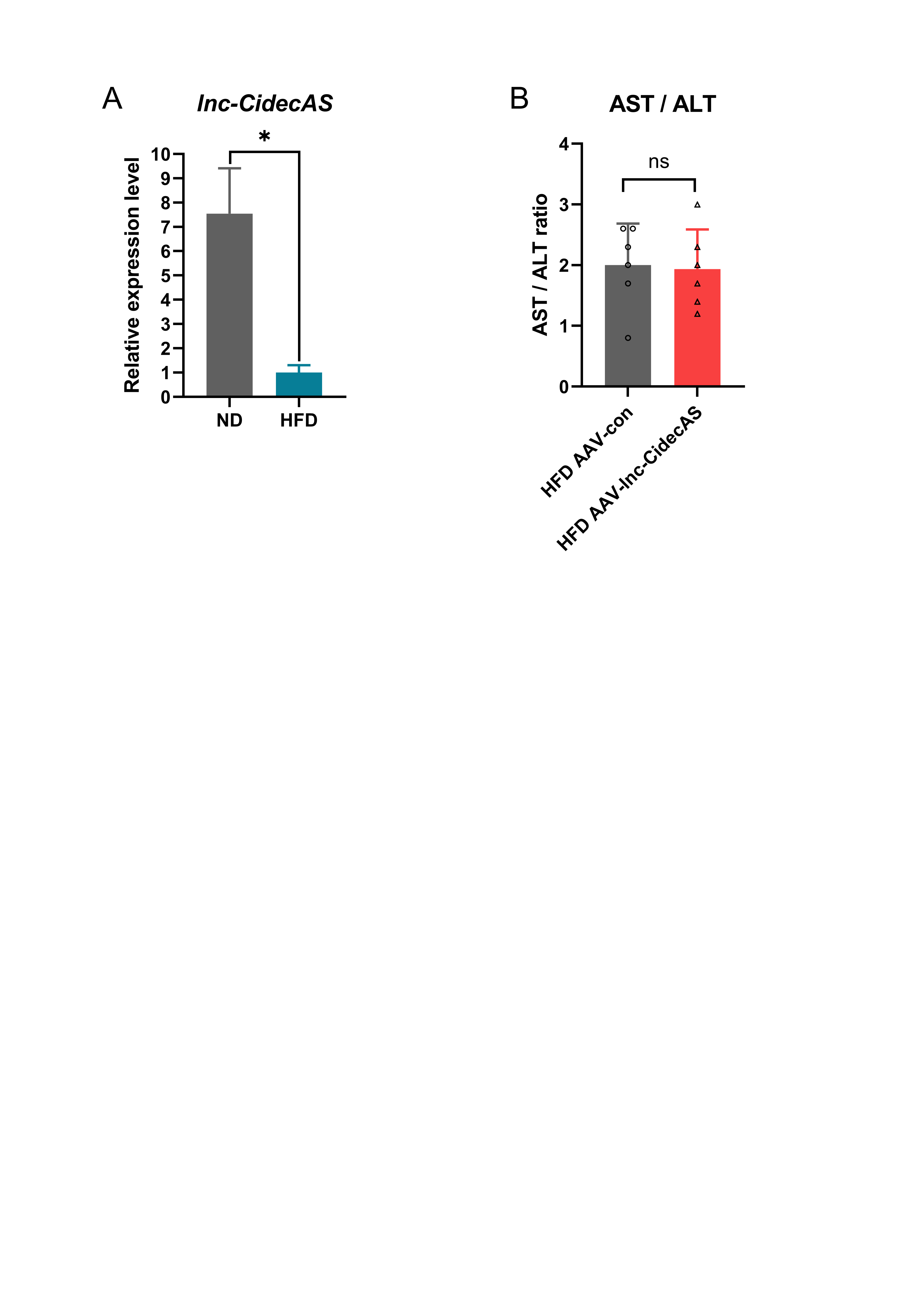

Supplement: Supplementary file 4 [file Image_2.TIF]
